# Mapping a leaf rust resistance gene *LrOft* in durum wheat Ofanto and its suppressor *SuLrOft* in common wheat

**DOI:** 10.3389/fpls.2023.1108565

**Published:** 2023-04-21

**Authors:** Xiangxi Zhuansun, Junna Sun, Nannan Liu, Shengnan Zhang, Huifang Wang, Zhaorong Hu, Jun Ma, Qixin Sun, Chaojie Xie

**Affiliations:** ^1^ Key Laboratory of Crop Heterosis and Utilization (Ministry of Education), China Agricultural University, Beijing, China; ^2^ Beijing Key Laboratory of Crop Genetic Improvement, Beijing, China

**Keywords:** durum wheat, leaf rust resistance, fine mapping, resistance suppressor, NB-ARC

## Abstract

Epidemics of leaf rust (caused by the fungal pathogen *Puccinia triticina* Erikss., *Pt*) raise concerns regarding sustainability of wheat production. Deployment of resistant cultivars is the most effective and economic strategy for combating this disease. Ofanto is a durum wheat cultivar that exhibits high resistance to *Pt* race PHT throughout its entire growing period. In the present study, we identified a leaf rust resistance gene in Ofanto and temporarily designated it as *LrOft*. *LrOft* was mapped to a 2.5 cM genetic interval in chromosome arm 6BL between Indel markers *6B6941* and *6B50L24*. During introgression of *LrOft* from Ofanto to common wheat it was observed that F_1_ plants of Ofanto crossed with Shi4185 exhibited leaf rust resistance whereas the F_1_ of Ofanto crossed with ND4503 was susceptible. In order to map the presumed suppressor locus, a Shi4185/ND4503//Ofanto three-way pentaploid population was generated and *SuLrOft* was mapped on chromosome arm 2AS. *SuLrOft* was mapped within a 2.6 cM genetic interval flanked by *2AS50L14* and *2AS50L6*. Fine mapping using 2,268 plants of the three-way cross narrowed the suppressor locus to a 68.2-kbp physical interval according to IWGSC RefSeq v1.1. Sequence analysis of genes in the physical interval revealed that *TraesCS2A02G110800* encoding an RPP-13-like protein with an NB-ARC domain was a potential candidate for *SuLrOft*.

## Introduction

Leaf rust, caused by *Puccinia triticina* (*Pt*), is a biotrophic foliar fungal disease of wheat that is more widespread globally than stem rust (*P. graminis* f. sp. *tritici*, *Pgt*) or stripe rust (*P. striiformis* f. sp. *tritici*, *Pst*) (Bolton et al., 2008). Early onset of leaf rust in wheat under favorable conditions generally reduces the thousand-grain weight and causes yield losses as high as 50% (Huerta-Espino et al., 2011). In China, leaf rust is a serious disease endangering wheat production, especially in the North China Plain, middle and lower reaches of the Yangtze River, southwest and northwest China (Liu and Chen, 2012). In recent years, the incidence of wheat leaf rust has been increased due to the climate conditions (Zhang et al., 2015; Zhang et al., 2020a; Zhang et al., 2020b). Genetically controlled disease resistance is the most economic and environmentally safe way to control leaf rust (Bariana et al., 2007; Singh et al., 2013). More than 80 leaf rust resistance genes (*Lr*) have been identified, many of which have been utilized in wheat breeding (Singh et al., 2013; Qureshi et al., 2018; Kumar et al., 2021; Xu et al., 2022). However, the occurrence of new virulent races can cause loss of effectiveness of resistance genes, and formerly resistant cultivars become susceptible. Therefore, it is necessary to search for new effective resistance sources.

Many of the leaf rust resistance genes were identified from wheat relatives (Assefa and Fehrmann, 2000), including tetraploid wheats *Triticum turgidum ssp. dicoccum*, *Triticum turgidum ssp. dicoccoides* and *Triticum turgidum ssp. durum* with the AABB genome. According to Herrera-Foessel et al. (2008), the gene *Lr14a* originated from cultivated emmer wheat cultivar Yaroslav was transferred to common wheat by McFadden (1930). *Lr53* and *Lr64* were derived from *T. dicoccoides* (Marais et al., 2005; Dadkhodaie et al., 2011) and *Lr23* was from durum cultivar Gaza (Watson and Stewart, 1956; Watson and Luig, 1961). Genes *Lr72* and *Lr79* were identified in durum cultivar Atil C2000 and landrace Aus26582, respectively (Qureshi et al., 2018; Kolmer et al., 2019).

The disease resistance genes in tetraploid wheat can be easily introduced into common wheat by direct crossing or backcrossing using common wheat as the recurrent parent. However, resistance genes derived from species with lower-ploidy may have reduced effectiveness or even become ineffective when introduced into high-ploidy species (Assefa and Fehrmann, 2000; Assefa and Fehrmann, 2004; Chen et al., 2013). Kerber and Green (1980) first reported suppression of stem rust in common wheat observing that removal of D genome chromosomes from the susceptible hexaploid wheat cultivar “Canthatch” (CTH) activated resistance to several *Pgt* races. The gene conditioning suppression was dominant, located in chromosome arm 7DL and named *SuSr-D1* (Suppressor of stem rust resistance 1, D-genome) following analysis of CTH nullisomic and ditelosomic stocks and EMS-derived mutants (Kerber and Green, 1980; Kerber, 1991). *SuSr-D1* encodes Med15b.D, a subunit of the Mediator Complex, a conserved protein complex in eukaryotes that regulates expression of protein-coding genes (Hiebert et al., 2020). Suppression seems to be a common phenomenon in wheat (Wilson and McMullen, 1997). Bai and Knott (1992) reported that some leaf rust resistance genes in durum wheat were suppressed in crosses with bread wheat. They found that a gene or genes on chromosome 3D of “Chinese Spring” (CS) suppressed resistance in three *T. dicoccoides* accessions; another gene or genes on chromosome 1D suppressed the leaf rust resistance in one of the three *T dicoccoides* accessions. Suppression of disease resistance can involve interaction of orthologous genes in hexaploid wheat (McIntosh et al., 2011). Nelson et al. (1997) found that the gene *Lr23* on chromosome 2BS in durum wheat Altar 84 was suppressed in certain synthetic lines by *SuLr23* on chromosome 2DS, and predicted that the latter was homoeologue of *Lr23*. Hanušová et al. (1996) reported that some lines carrying the Petkus rye chromosome arm 1RS failed to express the powdery mildew resistance gene *Pm8* known to be located in 1RS. It was later shown that *Pm8* was suppressed by some alleles of the orthologous wheat locus *Pm3* on chromosome 1AS (Hurni et al., 2014). Both the *Pm3* and *Pm8* alleles encode nucleotide-binding-leucine-rich repeat (NLR) resistance proteins and direct interaction of alleles of *Pm3* or *Pm8* caused interference/suppression of resistance (Stirnweis et al., 2014).

Italian durum cultivar Ofanto is highly resistant to leaf rust when inoculated by *Pt* race PHT at the seedling and adult stages. We crossed Ofanto with Chinese common wheat cultivars to transfer the leaf rust resistance of Ofanto into our common wheat breeding populations. While the resistance of Ofanto was effective in cross with common wheat cultivar Shi4185, it was not effective when cross was made with common wheat line ND4503, indicating the suppression of resistance of Ofanto. In this study, we analyzed the genetic basis of leaf rust resistance in Ofanto and also the suppression in crosses with ND4503. We mapped a dominant leaf rust resistance gene in Ofanto and its dominant suppressor in ND4503.

## Materials and methods

### Plant and pathogen materials

Italian durum wheat cultivar Ofanto is a spring cultivar that is resistant to leaf rust and powdery mildew diseases in Beijing, China. Its pedigree is Appulo/Valnova (De Vita et al., 2007). The susceptible durum wheat line Mo75 was provided by Prof. Xiao Chen (Institute of Crops, China Academy of Agricultural Science). We crossed Ofanto with Mo75 to generate F_1_ seeds and its derived F_2_ populations of 706 plants for genetic analysis. The pedigree of the elite common wheat cultivar Shi4185 is Zhi8.94/Baofeng7228//Shi84-7120. The common wheat line ND4503 was bred by China Agricultural University with the pedigree as ND3338/F390//Jingnong98-270. All the seeds of Ofanto, Mo75, Shi4185 and ND4503 are kept at China Agricultural University. We hypothesize that the lack of resistance in ND4503/Ofanto F_1_ is due to the effect of recessive gene action in Ofanto or suppressor in ND4503. To investigate the genetic basis of the susceptible F_1_ plants ND4503/Ofanto (6x/4x), we developed a three-way cross Shi4185/ND4503//Ofanto (6x/6x//4x) to create a mapping population that included 2537 plants (269 for genetic analysis, BSA analysis and primary mapping, 2268 for fine mapping). An F_1_ was first obtained by crossing Shi4185 by ND4503, and the resulting F_1_ was crossed with Ofanto. The seedling plants of the three-way Shi4185/ND4503//Ofanto population were used for leaf rust testing. The F_1_ plants of the three-way cross (AABBDD X AABB) were pentaploid and sterile. Susceptible common wheat line Xuezao was used as a check in all experiments.

Urediniospores of *Pt* race PHT were originally provided by the Institute of Plant Protection, Chinese Academy of Agricultural Sciences, Beijing and were subsequently propagated on a susceptible genotype. PHT was avirulent on Ofanto and virulent on durum wheat line Mo75, common wheat cultivars/lines Shi4185, ND4503 and Xuezao. The urediniospores were propagated in the greenhouse on the susceptible plants. In all experiments, a susceptible common wheat line Xuezao was used as a check for successful inoculation.

### Disease evaluation and statistical analysis

The phenotypes of both parents and Ofanto/Mo75 F_2_ and Shi4185/ND4503//Ofanto three-way progenies were evaluated at the seedling stage in the greenhouse. Wheat seeds were planted in 200-hole trays (10 × 20) at a density of one seed per hole in each tray and placed in a greenhouse at 15–20 °C. Approximately 15 days later, the seedlings with first leaves fully unfolded were inoculated; they were sprayed with a 1% aqueous solution of Tween-20® as surfactant followed by dusting with urediniospores and incubation in dark humidity chambers at 15 °C for 24 h before moving to a greenhouse maintained at 15–20 °C. Infection types (ITs) were evaluated 14 days after inoculation using a 0 to 4 scale (0 = hypersensitive flecks, 1 = small uredinia with necrosis, 2 = moderate size pustules with chlorosis, 3 = moderate-large size uredinia without necrosis or chlorosis, and 4 = large uredinia lacking necrosis or chlorosis) (McIntosh et al., 1995). ITs 0–2 were considered resistant, and the ITs 3–4 were considered susceptible. We tested 706 plants of Ofanto/Mo75 F_2_ population and 2537 plants of three-way cross Shi4185/ND4503//Ofanto mapping population. We rechecked the results of phenotypes two more times after the first disease evaluation at two-day intervals. A chi-squared analyses was performed on segregation results to confirm the goodness of fit of observed and predicted ratios. The χ^2^ analyses were executed in Microsoft Excel (version 2010) using the Bchitestˆ function to calculate χ^2^ and p-values.

### DNA extraction and quantification

After disease evaluation, leaf tissues of segregating populations and parents were collected and kept at -80 °C. Leaves were ground into powder in liquid nitrogen and DNA was extracted by the CTAB method (Maroof et al., 1994). DNA samples were quantified using a NanoDrop One spectrophotometer instrument (Nanodrop Technologies) and diluted to a working concentration of 30 ng/µl.

### Bulked segregant analysis with the SNP array

Bulked Segregant Analysis (BSA) was performed using the KPS Wheat 90K/660K Chip according to the Affymetrix Axiom 2.0 Assay Manual Workflow protocol provided by Compass Biotech Co. (CBC, Beijing) to identify SNPs associated with leaf rust response (Guan et al., 2019). To make the BSA analysis of the resistance gene in Ofanto by KPS Wheat 660K Chip, genomic DNA extracted from 20 resistant and 20 susceptible plants from the Ofanto/Mo75 F_2_ population were selected randomly and bulked in equal amounts to form resistance and susceptibility pools, respectively. For BSA analysis of the suppressor gene in ND4503, we selected 20 resistant and 20 susceptible plants from 269 plants of Shi4185/ND4503//Ofanto three-way population to make resistance and susceptibility pools, respectively. Markers polymorphic between the pools were then tested individually and confirmed across the mapping population.

The screened probes between pools were subjected to BLAST analysis to reveal their physical positions with respect to the CS reference genome sequence (IWGSC RefSeq v1.0). Next, the chromosomal segments enriched by these probes were analyzed. The workflow of genotype detection using the KPS Wheat 660K SNP array was similar to the 90K array described above.

### Resequencing of parental genomes

To improve the efficiency of marker development in resistance gene mapping, we resequenced Ofanto and Mo75 by double-end sequencing using the Illumina HiSeq2500 sequencing platform at Novogene Bioinformatics Company Ltd., NBC, Beijing (Li et al., 2020). All high-quality sequence reads were aligned to the durum wheat Svevo reference genome using the Burrows-Wheeler Aligner 0.7.15 program with default parameters (Li and Durbin, 2009; Maccaferri et al., 2019). DNA libraries of Shi4185 and ND4503 were prepared in the same way and aligned with sequence reads of the CS reference genome (IWGSC RefSeq v1.0). The Shi4185 re-sequencing data are available under NCBI Sequence Read Archive accession PRJNA476679 (https://downloads-qcif.bioplatforms.com/bpa/wheat_cultivars/cultivars/). We submitted the re-sequencing data of Ofanto, Mo75, and ND4503 in Library ID SUB12497795 with accessions SAMN32108327, SAMN32108328 and SAMN32108330.

### Marker development and genotyping

According to previously described methods, the insertion/deletion (InDel) variations between pairs of parents (i.e., Ofanto and Mo75, or Shi4185 and ND4503) in the target interval were chosen to design InDel markers (Chai et al., 2018). Based on IWGSC CS RefSeq v1.0, 24 polymorphic InDel markers were developed from polymorphisms between Shi4185 and ND4503 within the 56–149 Mb region of chromosome 2A. Primers were designed using primer3 v0.4.0 (http://bioinfo.ut.ee/primer3–0.4.0/) and CS IWGSC RefSeq v1.0 was employed to download sequences 200 bp upstream and downstream of the InDels. The 10 µL PCR system comprised 5 µL 2× TaqPCR StarMix, 1 µL of primers, 2 µL of 50–100 ng/µL DNA template, and 2 µL of H_2_O. For polymorphism detection, PCR products were separated by 3% agarose gel electrophoresis with TAE buffer and 10% non-denaturing polyacrylamide gel electrophoresis (PAGE) (Marklund et al., 1995).

Kompetitive Allele-Specific PCR (KASP) markers were designed from SNPs (Ramirez-Gonzalez et al., 2015; Fang et al., 2020) using Polymarker (http://www.polymarker.info/). Three KASP markers were developed from SNPs between Shi4185 and ND4503 to narrow down the suppressor locus. The reaction mixtures subjected to the TouchDown program comprised 2 µL of 100–200 ng/µL DNA template, 2 µL of 2× KASP master mixture, and 0.1 µL of primer mixture, totaling a volume of 4.1 µL. Fluorescent signals from the PCR mixtures were detected on a Real–Time Quantitative PCR instrument (Bio-Rad Laboratories Inc., USA). Sequences of the InDel and KASP markers are listed in **Table S1**.

### Genetic analysis and map construction

Markers polymorphic between resistant and susceptible parents were used to genotype Ofanto/Mo75 F_2_ plants and Shi4185/ND4503//Ofanto three-way plants. The leaf rust response data were used for linkage analysis in combination with PCR amplification results. Localization of markers and target gene was based on recombination between marker genotypes and disease phenotype. Genetic distances were calculated in centiMorgans (cM). JoinMap 4.1 was used to construct the initial linkage map. We used regression mapping algorithm (Stam, 1993; Van Ooijen and Voorrips, 2006) and Kosambi mapping function to calculate centiMorgans between markers (Kosambi, 1943). We chose the F_2_ population setting in Joinmap 4.1 to analyze the genetic linkage of *LrOft* and BC_1_ population setting for *SuLrOft*.

### Sequence analysis of candidate genes

The DNA sequences and approximately 500-bp upstream and downstream annotated gene sequences within the mapped interval amplified from Ofanto, Shi4185, and ND4503 using TKS Gflex™ DNA polymerase (TAKARA, Dalian), along with the corresponding primer pairs, are provided in **Table S1**. PCR products were separated in 1% agarose gels by electrophoresis and then sequenced at TsingKe Biological Technology Company, Beijing. Sequences were compared and analyzed using DNAMAN version 8.0 (Li et al., 2020). The SMART program (http://smart.embl-heidelberg.de/) was employed to predict the function of the candidate gene.

## Results

### Genetic analysis of leaf rust resistance in Ofanto

**Figure 1:**
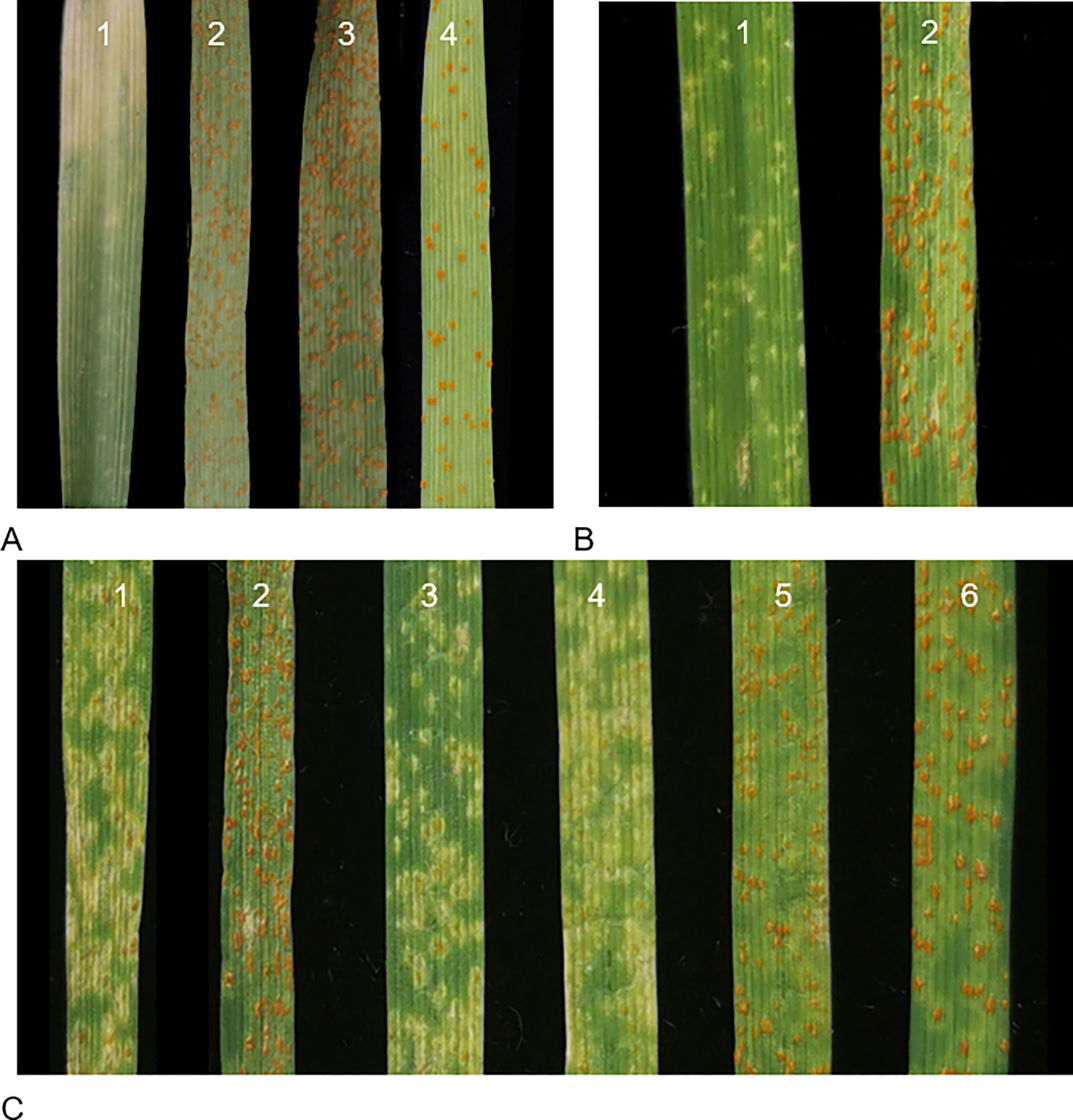
Seedling responses 15 days post-inoculation with the *Pt* race PHT. **(A)** Ofanto (1), IT 0;, (2) Shi4185 IT 4, (3) ND4503 IT 4, and (4) Mo75 IT 4; **(B)** F_2_ plants from cross Ofanto × Mo75, (1) IT 1, (2) IT 4; **(C)** F_1_ plants of (1) Ofanto/Shi4185 IT 1; (2) Ofanto/ND4503 IT 4; (3–4) resistant plants IT 1 and (5–6) susceptible plants IT 4 from the three-way cross.

Ofanto displayed a resistant IT 0; against *Pt* race PHT, whereas Shi4185, ND4503, and Mo75 were susceptible with IT 4 (**Figure 1A**). F_1_ seedlings from crosses Ofanto/Mo75 were resistant indicating that the leaf rust resistance in Ofanto was dominant. In the F_2_ population derived from the Ofanto/Mo75 cross, 510 were resistant and 196 susceptible, fitting the ratio of 3:1 (χ^2^ = 2.87, p > 0.05) (**Table 1**; **Figure 1B**). These results indicated that the leaf rust resistance in Ofanto was governed by a single dominant allele, provisionally designated as *LrOft*.

**Table 1:** Segregation of leaf rust resistance in the F_2_ populations from Ofanto/Mo75 and three-way population Shi4185/ND4503//Ofanto crosses.

		Number of seedlings			
Cross	Population	Resistant	Susceptible	χ^2^_(ratio)_	P-value
Ofanto/Mo75	F_2_	510	196	2.87_(3:1)_	>0.05
Shi4185/ND4503//Ofanto	Three-way F_1_	132	137	0.093_(1:1)_	>0.05

### Chromosomal location of the *LrOft* locus

**Figure 2:**
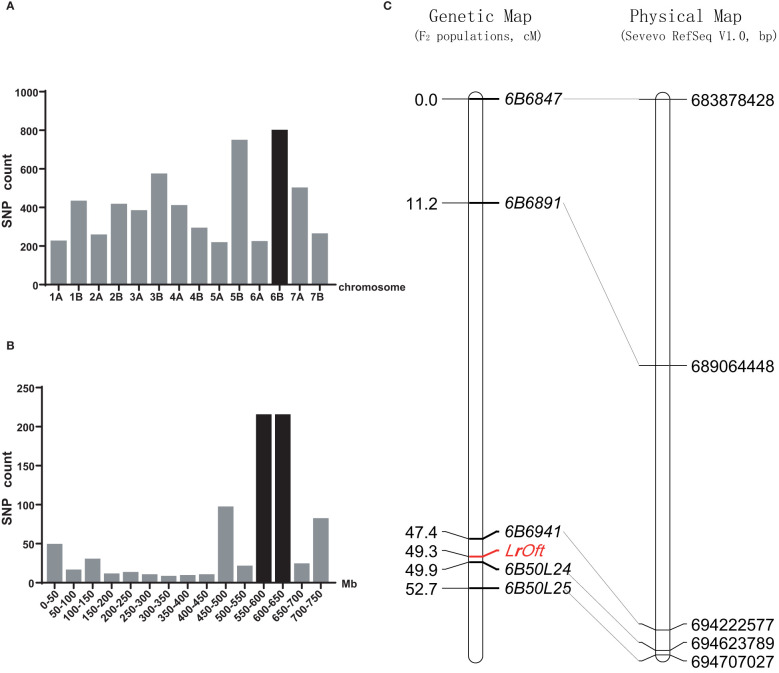
Chromosomal location of the *LrOft* locus. **(A)** Distribution of SNPs between the resistant and susceptible DNA pools from Ofanto/Mo75 F_2_ population on each chromosome; **(B)** distribution of candidate SNPs on chromosome 6B according to durum wheat Svevo RefSeq v1.0; **(C)** genetic linkage map of chromosome arm 6BL and the collinearity of the developed markers with their physical positions in the durum wheat Svevo RefSeq v1.0 sequence.

There were 8,904 SNPs between the resistant and susceptible pools constructed by Ofanto/Mo75 F_2_ population; 795 were anchored on chromosome 6B, which was the highest number among the 14 chromosomes (**Figure 2A**). According to the Durum Wheat Svevo RefSeq v1.0, 214 SNPs were enriched in the 550–650 Mb region of chromosome 6BL (**Figure 2B**), suggesting the resistance gene was located on the long arm of chromosome 6B.

To map *LrOft* more precisely we designed five InDel markers based on sequence variation in the candidate region according to re-sequencing data from the parents. InDel markers *6B6847*, *6B6891*, *6B6941*, *6B50L24*, *6B50L25* were confirmed to be polymorphic between the parents and pools and were used to genotype the 211 F_2_ plants from cross Ofanto/Mo75. A genetic linkage map of *LrOft* gene was constructed from the data, in which the *LrOft* locus was localized to a 2.5 cM genetic interval; 1.9 cM distal to *6B6941* and 0.6 cM proximal to *6B50L24* (**Figure 2C**).

### Identification of a suppressor of *LrOft* in common wheat

We checked the plants in the three-way population by InDel marker 2AS50L14 and 2AS50L6 to make sure it’s true three-way hybrid. When we evaluated the leaf rust resistance of 269 plants of Shi4185/ND4503//Ofanto three-way population, we found that 132 were resistant and 137 susceptible, fitting the ratio of 1:1 (χ2 = 0.093, p > 0.05) (**Figure 1C**; **Table 1**), suggesting that ND4503 contained a genetic factor for suppression of *LrOft*.

**Figure 3:**
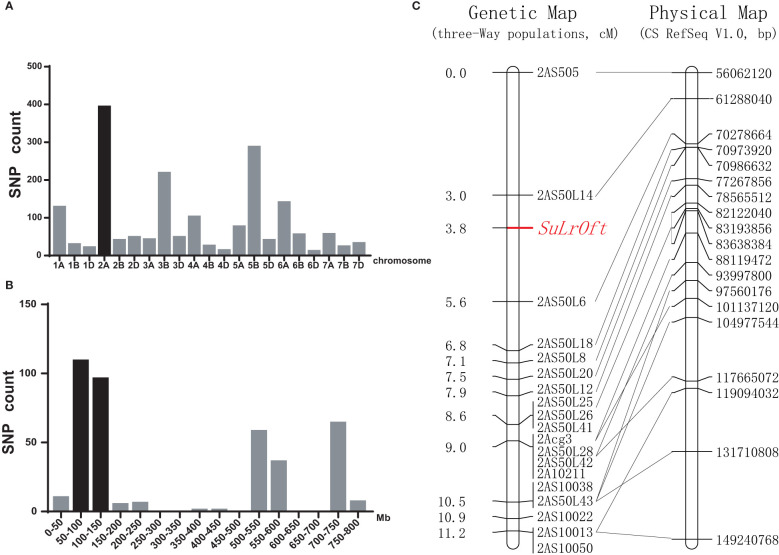
Chromosomal location of the *SuLrOft* locus. **(A)** Distribution of the candidate heterozygous/homozygous SNPs between the resistant and susceptible DNA pools from the Shi4185/ND4503//Ofanto three-way cross on each chromosome; **(B)** distribution of candidate SNPs on chromosome 2A according to the CS reference genome (IWGSC RefSeq v1.1); **(C)** genetic linkage map for chromosome arm 2AS and the collinearity of the developed markers corresponding to their physical positions in CS IWGSC RefSeq v1.0 sequence.

We identified 2,183 SNPs with heterozygous vs. homozygous variations between the resistant and susceptible pools in the three-way population that were genotyped with the 90K SNP array. Chromosome 2A contained the highest number (393) of SNPs (**Figure 3A**), and 305 of them were clustered in the 50–150 Mb region (**Figure 3B**), suggesting the genetic factor suppressing *LrOft* was located on chromosome 2A. Since *LrOft* was on chromosome 6B, non-homologous to chromosome 2A, it was likely that the suppressing factor in ND4503 was a suppressor of *LrOft*. We named it *SuLrOft*. Suppression of *LrOft* was conferred by heterozygous *SuLrOft* in the cross of Shi4185/ND4503//Ofanto (**Table S2**).

### Fine mapping of the *SuLrOft* locus

Two hundred and sixty-nine seedlings of the cross Shi4185/ND4503//Ofanto were genotyped using 19 InDel markers and a genetic map was constructed. The resultant genetic map of *SuLrOft* spanned 11.2 cM (*2AS505*-*2AS10013*) and the *SuLrOft* was delimited to a 2.6 cM interval flanked by markers *2AS50L14* and *2AS50L6*. According to the CS reference genome (IWGSC RefSeq v1.0), this interval corresponded to an approximate 9 Mb physical region (*2AS50L14* at 61288040 bp, and *2AS50L6* at 70278664 bp) (**Figure 3C**).

**Figure 4:**
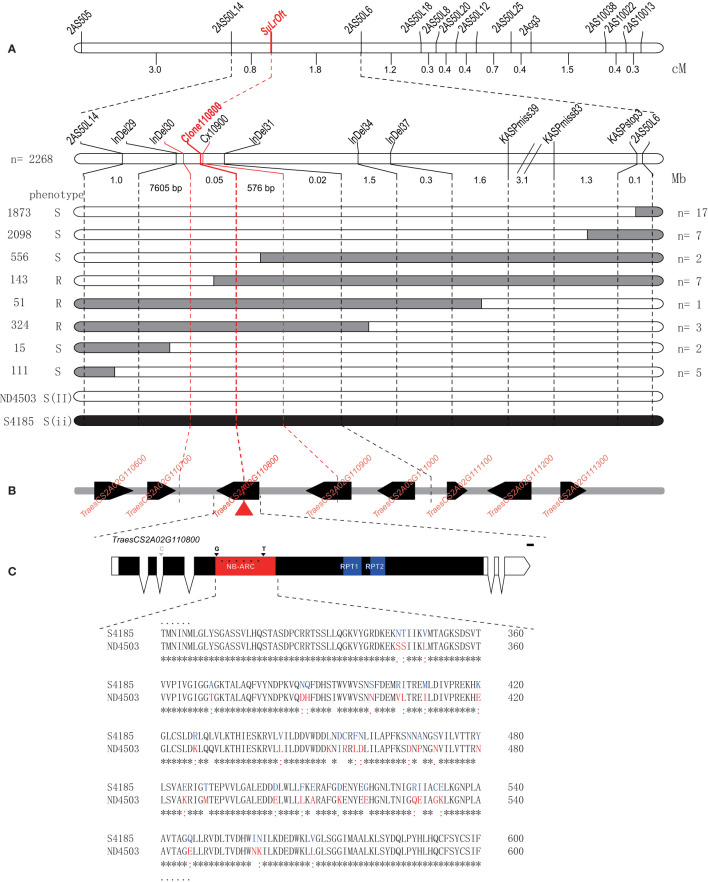
Fine mapping of the *SuLrOft* on chromosome 2AS. **(A)** Recombinant categories based on disease phenotype and molecular marker genotypes of plants in the three-way cross population. Black and white bars represent the homozygous segments from Shi4185 and ND4503, respectively; **(B)** annotated genes in the-fine mapped region according to the CS IWGSC RefSeq v1.0. The black boxes represent high-confidence genes, while arrows indicate the transcriptional orientation of each gene; **(C)** comparison of protein sequences in Shi4185 and ND4503.

To fine-map the genomic interval surrounding the *SuLrOft* locus another five InDel markers (*InDel29*, *InDel30*, *InDel31*, *InDel34*, and *InDel37*) and three KASP markers (*KASPmiss39*, *KASPmiss83*, and *KASPstop3*) were developed based on re-sequencing data of Shi4185 and ND4503 corresponding to the 9 Mb interval of the CS IWGSC RefSeq v1.0. After confirming polymorphisms between the parents, these eight new markers and the two closest flanking markers *2AS50L14* and *2AS50L6* were used to genotype an additional 2,268 F_1_ plants of cross Shi4185/ND4503//Ofanto; 67 recombinants between markers *2AS50L14* and *2AS50L6* were identified and 14 different recombinant genotypes were detected (**Table S3**). The allelic state for *LrOft* is heterozygous in each plant of the three-way population, including the 14 recombinants. Based on the genotypic and phenotypic data for the recombinants, the *SuLrOft* locus was delimited to the interval *InDel30*–*InDel31* (**Figure 4A**), corresponding to approximately 68.2 Kb in CS IWGSC RefSeq v1.1 (https://urgi.versailles.inra.fr/blast_iwgsc/). This interval contained three high-confidence and two low-confidence genes (**Figure 4B**).

### Determination of the candidate gene of *SuLrOft*

The re-sequencing data for Shi4185 and ND4503 revealed no variation in the two low-confidence genes (*TraesCS2A02G114800LC*, *TraesCS2A01G114900LC*). Among the three high-confidence genes, missense variations were found in the CDS of *TraesCS2A01G110800* and *TraesCS2A01G110900*, and there were two SNPs in 3’ UTR of *TraesCS2A01G111000*. *TraesCS2A01G110800* was annotated as a putative disease resistance RPP13-like protein and *TraesCS2A01G110900* was annotated as a putative disease resistance RGA4 protein. When we used primers *Cx10900* to amplify and sequence the SNP-containing intron segment (2A-62361245) in *TraesCS2A01G110900* in the eight recombinants between *InDel30* and *InDel31*, we found seven recombinants (**Table S4**) hence indicating that *TraesCS2A01G110900* is not the *SuLrOft* candidate. We cloned and sequenced *TraesCS2A01G110800* with primers *Clone110800* in the eight recombinants (**Figure S1**, **Table S1**) and the results showed that *SuLrOft* co-segregated with *TraesCS2A01G110800*, suggesting *TraesCS2A01G110800* as the most likely candidate gene (**Table S5**).

The SMART program (http://smart.embl-heidelberg.de/) was employed to predict the function of *TraesCS2A01G110800*, which encoded NB-ARC, RPT1 and RPT2 domains in the CDS region (**Figure 4C**). There were many sequence variations between Shi4185 and ND4503in *TraesCS2A01G110800*, most occurring in the NB-ARC domain, resulting in variations in amino acid sequences, with only one SNP in the second intron (**Figure 4C**).

## Discussion

Durum wheat is a primary gene pool for common wheat improvement. The Italian durum cultivar Ofanto has been demonstrated to be highly resistant to leaf rust in Beijing, China. Several leaf rust genes have been transferred to and utilized in common wheat breeding, including *Lr23* on chromosome arm 2BS, *Lr72* on arm 7BS, and *Lr79* on chromosome arm 3B (Herrera-Foessel et al., 2014; Qureshi et al., 2018). In the present study, we mapped resistance gene *LrOft* in durum cultivar Ofanto and located it on chromosome arm 6BL. Two genes, *Lr3* and *Lr9*, were previously localized on chromosome 6BL (Gupta et al., 2005). The *Lr9* resistance gene was transferred to wheat from *Aegilops umbellulata* (Sears, 1956; Sears et al., 1960). Schachermayr et al. (1994) developed the specific co-segregating STS marker *J13* for the detection of *Lr9*. The results of Blast analysis of the *Lr9* flanking markers sequences in IWGSC RefSeq v1.0 showed these sequences are specific in *Aegilops umbellulate*. However, our tests on Ofanto using *J13* primers indicated that Ofanto did not contain *Lr9* (data not shown). Herrera-Foessel et al. (2007) developed STS marker *Xmwg798* that co-segregated with *Lr3*. Ofanto was tested negative with this marker. Moreover, BLAST analysis results showed the *Xmwg798* was located at 690833283bp on chromosome 6B in Svevo RefSeq v1.0, whereas *LrOft* was localized in the 694.2 - 694.6Mb interval. Therefore, *LrOft* is likely located at a different position to *Lr3*. Herrera-Foessel et al. (2007) reported a previously unknown leaf rust resistance gene adjacent to *Lr3* in durum wheat line Camayo. The resistance gene *Lr_Camayo_* in Camayo was most likely derived from an Ethiopian landrace (Herrera-Foessel et al., 2007). According to De Vita et al. (2007), Ofanto was released in 1990 with the pedigree of Appulo/Valnova and does not seem to be related with Camayo. Since *Lr_Camayo_* was adjacent to *Lr3* and *Lr9*-cosegregating STS marker *Xmwg798* was at 690Mb on chromosome 6B, *LrOft* localized in the 694.2–694.6Mb interval might be allelic or closely linked to *Lr_Camayo_*. We can’t determine the relationship between *LrOft* and *Lr_Camayo_* in this study. Further studies are needed to determine their relationship.

The introgression of disease resistance genes from lower-ploidy wheat into hexaploid wheat can fail due to the presence of disease resistance suppression genes (Hanušová et al., 1996). In the present study, we found that the leaf rust resistance of Ofanto was inhibited when crossed with common wheat line ND4503. A three-way pentaploid population allowed us to map the suppressor *SuLrOft*. We fine-mapped *SuLrOft* in a 68.2-kb interval on the short arm of chromosome 2A. Nelson et al. (1997) reported suppression of *Lr23* on chromosome arm 2BS in a synthetic wheat line. In that example *SuLr23* was located in the homoeologous chromosome arm 2DS.

We found that the most likely candidate gene of *SuLrOft* was *TraesCS2A02G110800*, a putative disease resistance RPP13-like gene. Hurni et al. (2014) showed that powdery mildew resistance gene *Pm8* on chromosome arm 1RS from rye was suppressed by its wheat orthologue *Pm3* on chromosome arm 1AS. Both *Pm3* and *Pm8* encoded nucleotide-binding-leucine-rich repeat (NLR) resistance proteins, and some *Pm3* alleles interacted with *Pm8* to suppress resistance conferred by *Pm8* (Stirnweis et al., 2014). Nelson et al. (1997) also suggested that *SuLr23* might be specific for *Lr23* and orthologous to it. Studies are underway to prove the function of *TraesCS2A02G110800* as the candidate for *SuLrOft*. However, there is still a possibility that the sequence corresponding to *SuLrOft* is absent in the CS genomic sequence. Therefore, analysis of re-sequencing data based on the reference genome sequence is not sufficient to identify the target gene with certainty. Further research is needed to confirm the results in this study.

## Data availability statement

The datasets generated during and/or analyzed during the current study are available from the corresponding author on reasonable request.

## Author contributions

CX conceived the project. XZ performed the research. NL, JS, SZ and HW participated in field work. XZ constructed the linkage map and developed InDel markers. The first draft of the manuscript was written by XZ and all authors commented on previous versions of the manuscript. All authors contributed to the article and approved the submitted version.

## Supplementary material

Supplementary Figure 1 Sanger sequencing of *TraesCS2A02G110800* in Ofanto, Shi4185, and ND4503.
