# Supplementary figures and images for: Mapping a leaf rust resistance gene LrOft in durum wheat Ofanto and its suppressor SuLrOft in common wheat
Source: Front Plant Sci. 2023 Apr 21;14:1108565. doi: 10.3389/fpls.2023.1108565 (PMC10161252; doi:10.3389/fpls.2023.1108565)

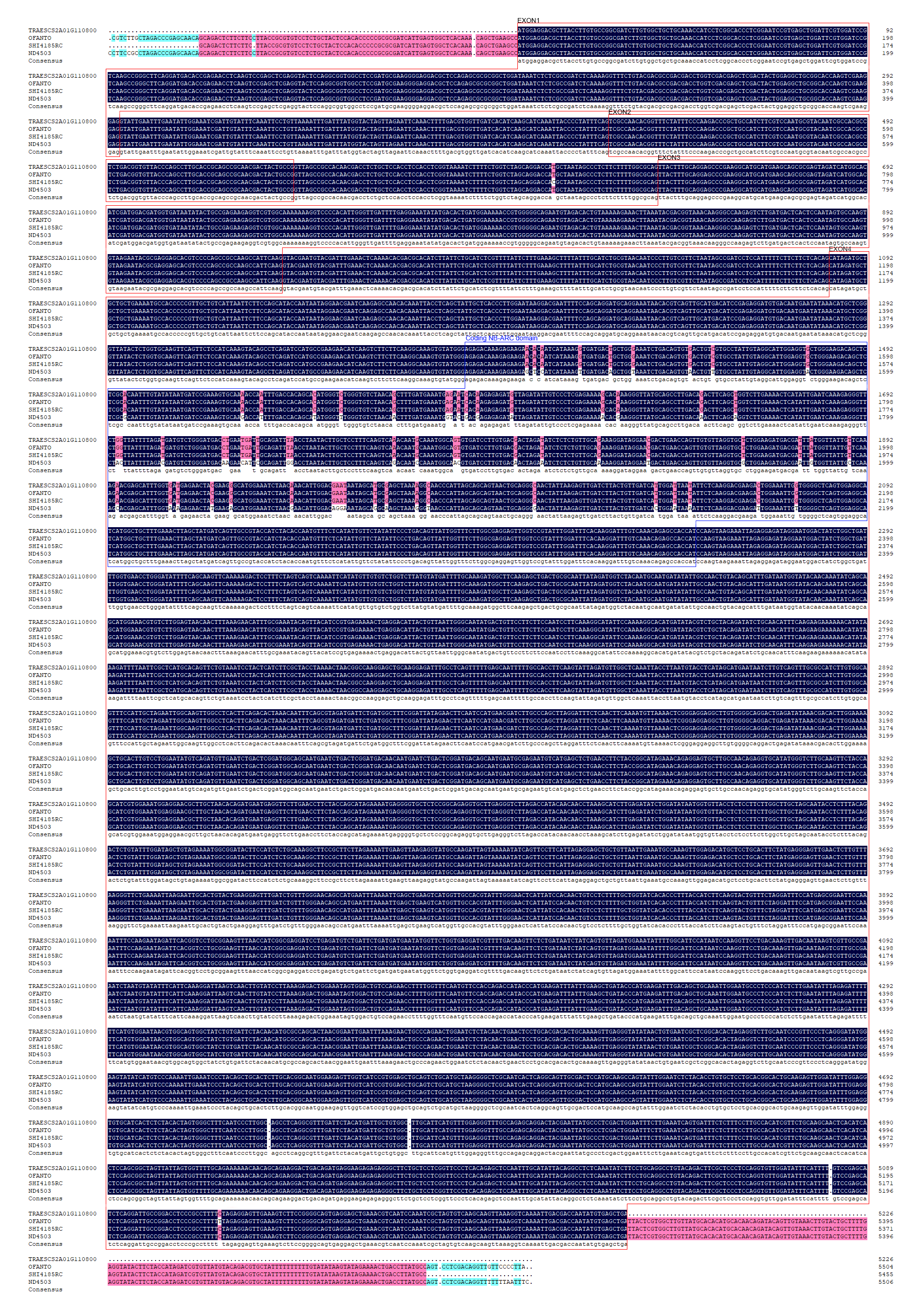

Supplement: Supplementary Figure 1 — Sanger sequencing of TraesCS2A02G110800 in Ofanto, Shi4185, and ND4503. [file Image_1.tif]
